# Undermining government tax policies: Common legal strategies employed by the tobacco industry in response to tobacco tax increases

**DOI:** 10.1016/j.ypmed.2017.06.012

**Published:** 2017-12

**Authors:** H. Ross, J. Tesche, N. Vellios

**Affiliations:** a Room 3.17, Economics of Tobacco Control Project, Southern Africa Labour and Development Research Unit, School of Economics, Middle Campus, University of Cape Town, Rondebosch, 7700, South Africa; b University of Maryland, University College, United States; c Room 3.19, Economics of Tobacco Control Project, Southern Africa Labour and Development Research Unit, School of Economics, Middle Campus, University of Cape Town, Rondebosch, 7700, South Africa

**Keywords:** Tobacco industry, Tobacco prices, Tobacco taxes

## Abstract

Effective tobacco tax increases reduce tobacco consumption, threatening the profitability of the tobacco industry. In response, the tobacco industry employs strategies to negate or minimize the full effects of tobacco tax increases. By interacting with various government agencies and non-governmental organizations we identified seven such strategies: stockpiling, changing product attributes or production processes, lowering prices, over-shifting prices, under-shifting prices, timing of price increases, and engaging in price discrimination and/or offering promotions. Each strategy is described in terms of the motivation for their employment, the consequences for tobacco use and tax revenue, and measures to counter them. Country case studies illustrate the successful execution of the strategies and possible government responses.

Many of the tobacco industry's responses to tobacco tax increases are predictable, since they are being employed systematically across countries. Governments can and should adopt appropriate measures to eliminate or reduce tobacco industry manipulation. This requires systematic data collection in order to monitor tobacco industry behavior.

## Highlights

Tobacco industry's responses to tobacco tax increases are predictable.
Governments can adopt appropriate measures to reduce tax avoidance.
Effective excise tax increases reduce tobacco use, decreasing prevalence of NCDs.
Systematic data collection by governments is needed to monitor industry behavior.

Raising the price of tobacco products by increasing taxes is one of the most effective measures to reduce tobacco use (International Agency for Research on Cancer, 2011). Since tobacco tax increases can decrease profitability, the tobacco industry has developed both legal and illegal strategies to mitigate their impact. While interacting with various government agencies and non-governmental organizations we identified seven legal strategies to reduce tobacco tax liability (stockpiling, changing product attributes or production processes, lowering prices, over-shifting prices, under-shifting prices, timing of price increases, engaging in price discrimination and/or offering promotions) and assess their impact on tobacco product prices, tax revenue and public health. Country case studies illustrate how these strategies have been used by the tobacco industry to undermine tobacco tax increases. The data for these examples came from the published literature and government agencies. We also offer solutions to limit these forms of tax avoidance.

1. **Stockpiling** (forestalling/front-loading) is over-supplying the market before a tax increase to pay the pre-tax-increase rate. It results in higher sales prior to a tax increase and lower sales following a tax increase as the oversupply is absorbed. This increases government tax revenues immediately before a tax increase, but reduces revenue immediately after, even though only temporarily. The industry tends to attribute the decrease in official sales and revenue immediately after a tax increase to higher illicit trade, and advocates for the return to a lower tax. Stockpiling can delay the impact of a higher tax on tobacco use if the products with the old tax are sold for the old, lower price. If prices are adjusted when the tax increase is announced, but before it comes into effect, consumption will decline, the industry/retailers will make extra profit, and tax revenue will decline until the tax increase comes into effect.

**Fig. 1:**
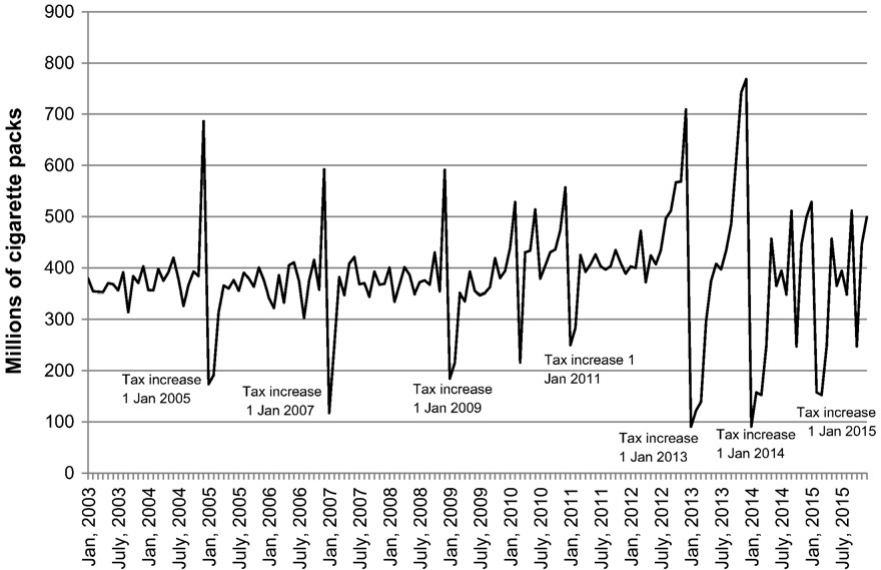
Volume of cigarette removals in Philippines (domestic production).

Source: Department of Finance - Fiscal Policy and Planning Office (DOF - FPPO) and the Bureau of Internal Revenue (BIR) of the Republic of the Philippines, 2016.

Fig. 1 shows that the volume of cigarette removals from warehouses in the Philippines (Department of Finance - Fiscal Policy and Planning Office (DOF - FPPO) and the Bureau of Internal Revenue (BIR) of the Republic of the Philippines, 2016) increases in anticipation of tax increases and decreases immediately after the tax change as the industry waits for the lower-taxed cigarettes to be sold. The government records showed inflated tax revenue before the tax increases followed by a drop afterwards (Quimbo et al., 2012, Kaiser et al., 2016). The front-loading was addressed during 2014 resulting in less revenue fluctuation in 2015 (Manila Bulletin, 2015).

Governments can prevent stockpiling by regulating how quickly old stock must be sold, limiting the quantity of tax stamps/products released to the market in the months prior to a tax increase, banning sales of products with old tax stamps, issuing stamps valid only for one fiscal year (Pederson et al., 2014), or making wholesalers responsible for paying the difference between the old and the new tax rate on the day the new tax is effective (so called floor tax) (Pederson et al., 2014).

**Fig. 2:**
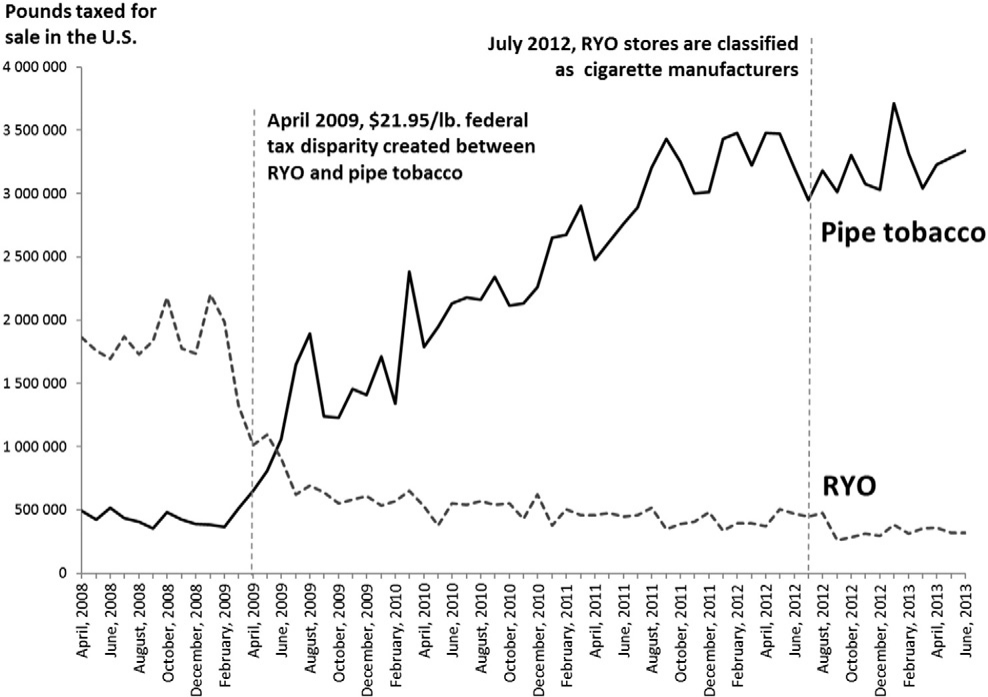
US sales of RYO and pipe tobacco 2008–2013.

Source: Tynan MA, Morris D, Weston T. Continued implications of taxing roll-your-own tobacco as pipe tobacco in the USA. Tob Control. 2015;24(e2):e125-e7. http://dx.doi.org/10.1136/tobaccocontrol-2013-051531.

2. **The tobacco industry can change product attributes** (e.g., weight, length) **or production processes** if different tobacco products are subject to different tax increases. This strategy has a negative impact on both expected revenue and public health outcomes. Fig. 2 demonstrates the impact of the industry relabeling of roll your own (RYO) as pipe tobacco in the US in 2009 when the government tax increase favored pipe tobacco.

Similarly, Indonesia's complex tax system favoring smaller scale producers motivated tobacco companies to split operations into smaller scale facilities (Euromonitor International, 2013). Imposing comparable tax rates on substitutable tobacco products, regardless of their characteristics, prices or production process, can prevent this form of tax avoidance (Curti et al., 2015).

3. **Lowering producer prices** on some or all products in an ad valorem tax regime reduces tax payments. Even though lower prices reduce profit margins, the overall profit might not decline if lower prices increase sales. Reducing prices of selective brands may also help to retain price sensitive customers. This will minimize the impact of a tax increase on sales/consumption and lower tax revenue.

In 2012, Senegal increased tobacco tax from 40% to 45% and from 20% to 40% of retail price on premium and economy brands, respectively. In response, Philip Morris International reduced the price of Marlboro from US$1.20 to US$0.79 per pack to reclassify it from premium to economy, thereby completely avoiding the tax increase (Senegal, 2011). Levying a uniform specific tax on all tobacco products/brands would prevent this type of tax avoidance.

4. **Over-shifting a tax increase** raises retail prices more than the tax increase. Higher profit margins compensate the industry for the reduction in sales due to higher tax while the government can be blamed for the entire price increase. Over-shifting can occur selectively, e.g. on higher priced brands, since the demand for them is usually less price-sensitive. Over-shifting at all price levels has a positive impact on public health since it suppresses demand, but results in less than predicted tax revenue. In South Africa, for example, inflation-adjusted tax increased by 377% from 1994 to 2010. During the industry's increased its net-of-tax price by 173%. This increased both tax revenue and the industry's profitability despite lower sales. This industry behavior indicates that the market can absorb higher prices, and therefore higher taxes.
5. **Under-shifting tax increases** is usually a temporary attempt to preserve sales as the industry absorbs a part of the tax increase, usually on low price brands, to retain price-sensitive consumers. This reduces the public health impact of tax increases due to a smaller than expected reduction in demand, but increases the tax revenue above the expectation due to larger than expected sales.

From January 2007 to January 2008, the inflation-adjusted cigarette excise tax in Ukraine rose by 6%, yet real cigarette prices fell by 11% (Ross et al., 2012) as the industry absorbed the small tax increase to keep prices and the demand stable. After more significant tax increases in 2009, the tobacco industry began to over-shift increasing its own price exclusive of tax by 39% from January 2009 to December 2010.

Setting a minimum tax floor can limit the industry's under-shifting, because it guarantees that a product cannot be sold for less than the minimum amount. This will enhance the effectiveness of tax as a public health tool.

6. **Strategic timing of a price increase** allows the customers to adjust gradually to higher prices, which may result in an overall smaller decrease in tobacco demand compared to a situation when prices are increased in one step (International Agency for Research on Cancer, 2011). If prices are increased before the tax increase goes into effect, the industry collects extra profit until the date of the tax implementation, and consumption and tax revenue will drop during this time. The temporary revenue loss is reversed once the new tax is implemented, but the impact of the tax increase on consumption will be reduced since the demand has already responded to new prices before the tax increase. The industry may exploit this by claiming that tax increases only affected tax revenue, but not tobacco demand, and therefore label it a failure from a public health perspective. Delaying a price increase until after a tax change will result in temporarily higher than expected tax revenue, no initial change in consumption and a temporary decrease in profit.

In February 2009, the USA increased cigarette excise tax from US$ 0.39 to US$ 1.01 per pack effective April 1, 2009. In anticipation of the tax increase, Philip Morris USA raised prices of both Marlboro and its other brands by US$ 0.71 and US$ 0.81 cents a pack in early March 2009, respectively, thus over-shifting the tax increase of US$ 0.62 before it went into effect (Adelman et al., 2009).

Governments should monitor tobacco product prices before and after tax changes in order to assess industry strategies and interpret sales and revenue data correctly to prevent industry misinformation about the market response to higher taxes.

7. **Price discrimination and/or price-related promotions** minimize the loss of price-sensitive customers after a price increase by offering discounts, retailer rebates, or adding value (e.g. gifts) to the purchase. This strategy, which is particularly effective for lower income groups due to their higher price-sensitivity, will contribute to smoking-related inequalities (International Agency for Research on Cancer, 2011). The tax increase will have a lower impact on consumption while the tax revenue will exceed expectations, particularly under a specific tax regime.

In the USA, Philip Morris USA contacted its customers, targeting women, youth and other price-sensitive consumers, with a coupon offer for buying cigarettes below the retail price right before the 2009 cigarette excise tax increase. In the UK, the industry responded to annual tax increases by launching new ultra-low price (ULP) brands in 2006 and keeping their real prices constant over time. As the price gap between the ULP and other brands widened, the ULP's market share doubled in 3 years (Gilmore et al., 2013).

Setting a minimum tax floor and/or banning price-related promotions/discounts will limit the industry's price discrimination. Governments should monitor brand proliferation (e.g. launch of cheap brands) in order to assess industry strategies and interpret the market data correctly.

## Discussion

The seven strategies discussed in this essay are often combined to hinder an effective implementation of tobacco tax policies, yet are rarely discussed in the literature. Governments have the power to regulate the industry and can use the Article 6 Guidelines of World Health Organization's Framework Convention on Tobacco Control to control these tax avoidance practices (World Health Organization, 2014). An effective tax administration requires monitoring and analyzing the industry behavior so that authorities can respond quickly and effectively. This will enhance their tax collection and improve public health by increasing the effectiveness of tobacco excise taxes.

The limitation of this study is that it is not a systematic literature review and the case studies were selected based on their ability to demonstrate a particular behavior. Since these types of misconduct are related to tax administration, they are usually described in internal government documents rather than in the scientific literature.

## Conflict of interest

None.
